# Clinical efficacy of Radix Arnebiae oil in patients undergoing incision and drainage of perianal abscess and its effects on pyroptosis

**DOI:** 10.3389/fcimb.2026.1945950

**Published:** 2026-09-11

**Authors:** Cheng Zhao, Er-Wei Cai, Wei-Juan Wang

**Affiliations:** 1 The Second Department of Anorectal, The Affiliated People’s Hospital of Fujian University of Traditional Chinese Medicine, Fuzhou, Fujian, China; 2 Department of Supervisor Nurse, The Affiliated People’s Hospital of Fujian University of Traditional Chinese Medicine, Fuzhou, Fujian, China

**Keywords:** clinical efficacy, pyroptosis, Radix Arnebiae oil, status post incision and drainage of perianal abscess, wound healing

## Abstract

**Objective:**

The aim of this study was to assess the clinical efficacy of Radix Arnebiae oil (RAO) in patients undergoing incision and drainage of perianal abscess and to investigate its effects on pyroptosis-related factors, thereby providing evidence to support its clinical application and offering insights into its potential mechanism of action.

**Methods:**

Ninety patients were randomly assigned to three groups (n = 30 per group) using a random number table: the RAO group, the Moist Exposed Burn Ointment (MEBO) group, and the petrolatum group. Beginning on postoperative day (POD) 1, the assigned topical agent was applied to the wound in each group and continued until complete wound healing. Pain and wound exudate scores were assessed on PODs 3, 7, and 14. In addition, wound healing rates and healing duration were recorded. Protein expression levels of caspase-1, interleukin (IL)-1β, and IL-18 were assessed using enzyme-linked immunosorbent assay (ELISA) and immunohistochemical (IHC) staining.

**Results:**

Treatment with RAO significantly alleviated postoperative pain and reduced wound exudation following incision and drainage of perianal abscess. On POD 14, the corresponding scores in the RAO group were significantly lower than those in the MEBO and petrolatum groups (p < 0.05). The RAO group demonstrated a higher wound healing rate and a significantly shorter healing duration than the MEBO and petrolatum groups (both p < 0.05). The overall clinical efficacy was significantly greater in the RAO group than in the other two treatment groups (p < 0.05). ELISA findings on POD 14 showed significantly lower protein expression levels of caspase-1, IL-1β, and IL-18 in the RAO group than in the petrolatum and MEBO groups, with statistically significant differences (all p < 0.05). Consistent with these findings, IHC staining demonstrated reduced expression of these proteins in the RAO group compared with the MEBO and petrolatum groups.

**Conclusion:**

RAO alleviates postoperative pain, reduces wound exudation, and promotes wound healing in patients undergoing incision and drainage of perianal abscess. Its mechanism of action may be associated with the regulation of pyroptosis-related mediators, including caspase-1, IL-1β, and IL-18.

## 1 Introduction

Anorectal abscess, commonly referred to as perianal abscess in clinical practice, is a common condition encountered in the anorectal surgery and is characterized by an acute onset. The condition predominantly affects young and middle-aged adults aged 20–40 years and accounts for approximately 8%–25% of anorectal diseases (Gaertner et al., 2022; Sun et al., 2023). Currently, surgical intervention remains the primary treatment modality. However, due to the unique anatomical characteristics of the perianal region, wounds following incision and drainage of perianal abscess are typically left open, resulting in substantial wound exudation. These wounds are frequently exposed to direct fecal contamination. Pain during dressing changes and anxiety related to defecation may adversely affect patient well-being and quality of life. Effective postoperative pain management and promotion of wound healing are of considerable clinical importance. Radix Arnebiae oil (RAO) is a proprietary preparation routinely used in the Department of Anorectal Surgery (Minyao Zhizi Z06106033). It has been widely applied in the postoperative management of anorectal wounds and has demonstrated favorable clinical efficacy by significantly alleviating symptoms following incision and drainage of perianal abscess and promoting wound healing.

## 2 Materials and methods

### 2.1 Clinical data

#### 2.1.1 Diagnostic criteria

The diagnostic criteria were established in accordance with the Chinese Expert Consensus on the Clinical Diagnosis and Treatment of Perianal Abscess, issued by the Anorectal Physicians Branch of the Chinese Medical Doctor Association (Guidelines Working Committee of the Proctology Branch of China Medical Doctor Association, 2018). According to the Guidelines for the Diagnosis and Treatment of Common Diseases of Coloproctology in Traditional Chinese Medicine (TCM), perianal abscess with the pattern of exuberant heat-toxin is characterized by the following principal clinical manifestations: severe swelling and pain in the perianal region persisting for several days; palpable fluctuance at the affected site with pus obtained on aspiration; chills, fever, poor sleep, dry mouth, constipation, and dysuria; as well as a red tongue with a yellow coating accompanied by a wiry-slippery or wiry-rapid pulse (China Association of Traditional Chinese Medicine, 2012).

#### 2.1.2 Inclusion criteria

Participants meeting all of the following criteria were eligible for enrollment: (1) Fulfilment of the diagnostic criteria for perianal abscess. (2) No documented history of perianal morphological or functional abnormalities and no history of surgery for other anorectal disorders. (3) Age between 18 and 60 years. (4) Undergoing incision and drainage of perianal abscess under spinal or general anesthesia. The study was approved by the Institutional Review Board (IRB) of the hospital (IRB approval number: 2024-057-02), and written informed consent was obtained from all participants prior to enrollment.

#### 2.1.3 Exclusion criteria

Participants meeting any of the following criteria were excluded: (1) Severe cardiovascular or cerebrovascular disease, or hepatic or renal dysfunction; (2) Infectious diseases, including pulmonary tuberculosis, hepatitis B, or sexually transmitted diseases; (3) Confirmed diagnosis of inflammatory bowel disease; (4) Metabolic diseases that could impair wound healing, such as diabetes mellitus; (5) Pregnancy or lactation; and (6) Presence of concomitant anorectal diseases.

#### 2.1.4 Baseline characteristics

A total of 90 inpatients treated in the Second Department of Anorectal Surgery at the Affiliated People’s Hospital of Fujian University of Traditional Chinese Medicine between March and August 2025 were enrolled after obtaining approval from the hospital Ethics Committee. The participants were randomly assigned to one of three groups (n = 30 per group): the RAO group, the MEBO group, and the petrolatum group. The randomization sequence was generated using a random number table. Specifically, 90 random numbers corresponding to the enrolled subjects were generated, and grouping cards were prepared in advance according to the enrollment order (1 to 90). Each grouping card was placed in a sequentially numbered, sealed, opaque, and identically appearing leather envelope to ensure allocation concealment. When a subject was enrolled, the researcher responsible for recruitment opened the corresponding numbered envelope in the order of enrollment to obtain the group assignment and then implemented the allocated intervention. This procedure ensured adequate allocation concealment and minimized selection bias. Baseline characteristics, including sex and age, were comparable among the three groups (all p > 0.05; **Table 1**).

**Table 1:** Baseline demographic characteristics of the study groups.

Group	Sex (*n*)		Age (x̅*±s*), years
	Male	Female	
RAO	18	12	39.13 ± 13.39
MEBO	23	7	39.70 ± 11.56
Petrolatum	19	11	35.70 ± 10.08
	χ^2^=2.10		F=1.016
	*P=*0.350		*P*=0.366

No significant differences in sex distribution or age were observed among the three groups, indicating comparable baseline characteristics.

### 2.2 Methods

#### 2.2.1 Treatment protocol

Routine postoperative care was provided to all patients on the day of surgery. Following each bowel movement and during daily wound cleansing, the assigned topical treatment (RAO, MEBO, or petrolatum) was applied evenly to the wound surface according to group allocation. RAO is a proprietary herbal oil preparation routinely used in our department (Minyao Zhizi Z06106033). The qualitative composition of the formulation includes Radix Arnebiae (sovereign herb), Rhizoma Coptidis and Radix et Rhizoma Rhei (minister herbs), and Gypsum Fibrosum, Rhizoma Bletillae, and Borneolum Syntheticum (assistant and courier herbs). It is prepared by combining these crude herbs, which are then macerated in tea oil at room temperature (25 ± 2 °C) for 14 days to allow adequate extraction of active constituents. The mixture is subsequently filtered through a 200-mesh sieve to obtain a clear, reddish-brown medicinal oil. The final product is manufactured under the Good Manufacturing Practice (GMP) standards of our hospital’s pharmaceutical preparation unit. Batch-to-batch consistency is ensured through routine quality control tests, including relative density (0.910–0.935), acid value (≤ 2.0 mg KOH/g), and identification tests for the characteristic compounds of Radix Arnebiae (e.g., shikonin derivatives) and Rhizoma Coptidis (e.g., berberine) by thin-layer chromatography (TLC), in accordance with the Chinese Pharmacopoeia standards. The product registration number (Minyao Zhizi Z06106033) certifies that it has met all regulatory quality requirements approved by the local drug administration authority. This oil is not a chemically defined ‘free oil’ but rather a complex herbal extract in an oil base. While the full quantitative formula is protected intellectual property, detailed technical information and reference samples of RAO are available to qualified researchers upon reasonable request to the corresponding author, subject to a standard material transfer agreement (MTA), to facilitate independent validation and replication studies. The final product is applied directly and evenly onto the wound surface once daily after routine wound cleansing.

Due to the marked differences in dosage form, odor, and appearance among the three topical preparations (RAO, MEBO, and petrolatum), double-blinding (blinding of both participants and healthcare providers) was not feasible. To minimize assessment bias, however, we implemented single-blinding for outcome assessors. All outcome measures—including pain scores, wound exudate scores, and wound area measurements—were independently evaluated by dedicated assessors who were unaware of the subjects’ group allocation. These assessors did not participate in the randomization, intervention, or dressing change procedures. After the assessments were completed, the data were transferred to a separate individual for data entry and analysis to further reduce information bias.

#### 2.2.2 Outcome measures

1. Pain score: Pain severity was evaluated according to the Guiding Principles for Clinical Research on New Drugs of TCM (Trial Edition, 2002) and adapted to the clinical context. Pain and wound exudate scores were assessed on PODs 3, 7, and 14. The pain score was assessed and assigned by professional medical personnel (rather than self-reported by the patients) based on the subject’s pain condition and clinical presentation, according to the criteria described below. The scoring criteria was as follows: 0 points, no pain at the incision site; 1 point, mild incisional pain that was tolerable and did not affect sleep; 2 points, moderate to severe incisional pain that affected sleep, but could was effectively relieved with a diclofenac sodium suppository; and 3 points, persistent, severe, and intolerable incisional pain associated with marked sleep disturbance, requiring combination therapy with bucinnazine due to inadequate analgesia provided by a rectal diclofenac sodium suppository.
2. Wound exudate score: Wound exudation was quantified according to the number of gauze layers saturated with exudate: 1 point, ≤ 2 layers saturated; 2 points, > 2 to ≤ 4 layers saturated; 3 points, > 4 to ≤ 6 layers saturated; and 4 points, > 6 to ≤ 8 layers saturated. For subsequent assessments, one additional point was assigned for every additional two layers of gauze saturated with exudate. The sterile gauze pads used in the study were manufactured by Yadu Medical Technology Co., Ltd. (75 mm × 75 mm, 8 layers).
3. Wound healing rate: The wound healing rate was calculated on postoperative days (PODs) 14, 21, and 28.

The wound measured on POD 1 was defined as the baseline wound area. Subsequent measurements were obtained on PODs 14, 21, and 28. Wound area was determined by multiplying the maximum length by the maximum width measured with a ruler. Following the onset of epithelialization, the advancing epithelial margin was used as the measurement boundary.


Wound area=maximum length×maximum width.

Wound healing rate=(initial wound area−current wound area)/initial wound area×100%.

4 Wound healing time: Wound healing time was defined as the interval from POD 1 to complete wound healing and was recorded for each participant.

#### 2.2.3 Assessment of caspase-1, interleukin-1β, and interleukin-18 in wound tissue

On PODs 3, 7, and 14, granulation tissue specimens were collected from participants in the three groups for enzyme-linked immunosorbent assay (ELISA) and immunohistochemical (IHC) analyses. The principal reagents and instruments used in this study included a Caspase-1 ELISA Kit (Catalog No. MM-13398H1), an IL-1β ELISA Kit (Catalog No. MM-13354H1), an IL-18 ELISA Kit (Catalog No. MM-13375H1), and a ready-to-use two-step rabbit/mouse IgG IHC Detection Kit (Boster Biological Technology Co., Ltd., Catalog No. 18B13B04).

To ensure consistency and minimize tissue heterogeneity, all biopsies were performed at predetermined postoperative time points (PODs 3, 7, and 14) using a standardized protocol. Specifically, all samples were taken from the central area of the wound, carefully avoiding the wound edges, surrounding normal skin, and any visible necrotic tissue. The sampling location was demarcated prior to each biopsy to ensure that specimens from different groups and time points were obtained from comparable anatomical sites. Immediately after collection, visible necrotic, fibrous, and adipose tissues were meticulously removed, retaining only fresh granulation tissue. Each specimen was further trimmed under a stereomicroscope to eliminate residual non-target tissues. For molecular assays, the entire granulation tissue sample was thoroughly homogenized to average out any potential regional differences within the wound. These procedures were consistently applied across all groups and time points.

(1) Measurement of caspase-1, IL-1β, and IL-18 protein levels by ELISA.

Tissue specimens were transferred to a glass homogenizer and homogenized in ice-cold phosphate-buffered saline (PBS) at a ratio of 5 mL PBS per gram of tissue. Following centrifugation, the supernatant was collected for analysis. All procedures were performed in accordance with the instructions provided by the manufacturer.

(2) Detection of caspase-1, IL-1β, and IL-18 by immunohistochemistry.

Tissue specimens were fixed, paraffin-embedded, sectioned, and subjected to IHC staining. All procedures were performed in accordance with the instructions provided by the manufacturer. The stained sections were examined and photographed using light microscopy.

### 2.3 Statistical analysis

The sample size was estimated using the formula for comparison of multiple means in a completely randomized design:


$$n=\frac{\varphi^{2}(\sum \sigma_{i}^{2}/k)}{\Sigma {\left(\mu_{i}-\mu \right)}^{2}/(k-1)}$$

where n is the required sample size per group, σ_i_ is the standard deviation of each population, μ_i_ is the mean of each population, μ̄ = Σμ_i_/k, and k is the number of groups. Referring to previous similar studies, with α = 0.05 and β = 0.1, the required sample size was at least 28 cases per group. Accounting for a 10% dropout rate, 30 cases per group (90 cases in total) were ultimately enrolled.

Statistical analyses were performed using SPSS version 26.0 software. Continuous variables were expressed as mean ± standard error of the mean. For repeated measures across time points, a repeated measure analysis of variance (ANOVA) was employed to evaluate the main effects of group and time, as well as their interaction. If a statistically significant interaction effect was found, simple effects analysis was conducted, and the Bonferroni method was used to correct for multiple comparisons. One-way analysis of variance or the Kruskal-Wallis test was used for between-group comparisons at individual time points, as appropriate. Categorical variables were analyzed using the chi-square test, while ordinal data were analyzed using the rank-sum test. A two-sided p value < 0.05 was considered statistically significant.

## 3 Results

### 3.1 Comparison of pain and wound exudate scores among the three groups at different postoperative time points

No statistically significant differences in pain or wound exudate scores were observed among the three groups on POD 1 (p > 0.05). On POD 14, both pain and wound exudate scores were significantly lower in the RAO group than in the MEBO and petrolatum groups (both p < 0.05). These findings indicate that RAO may alleviate postoperative pain and wound exudation following incision and drainage of perianal abscess. In all three groups, pain and wound exudate scores on POD 7 were higher than those on POD 1, which may have been associated with the postoperative inflammatory response, ligature slippage, and increased cellular metabolic activity following incision and drainage of perianal abscess. Detailed results are presented in **Table 2**.

**Table 2:** Comparison of pain and wound exudate scores among the three groups on PODs 3, 7, and 14 (x̅ *± s*).

Group	*n*	Time point	Pain score	Wound exudate score
RAO	30	3d	2.23 ± 0.72	1.47 ± 0.63
		7d	2.57 ± 0.89	2.57 ± 0.57
		14d	0.50 ± 0.73^Δ^	1.03 ± 0.18^*^
MEBO	30	3d	2.00 ± 0.64	1.57 ± 0.62
		7d	2.43 ± 0.89	2.87 ± 0.73
		14d	1.03 ± 0.81^Δ^	1.37 ± 0.49^*^
Petrolatum	30	3d	2.17 ± 0.64	1.27 ± 0.45
		7d	2.40 ± 0.72	2.90 ± 0.80
		14d	1.23 ± 0.89^Δ^	1.57 ± 0.50^*^
F			6.485	12.383
*P*			0.002	0.000

^Δ^*p*<0.05. *p* < 0.05 for RAO group vs. MEBO and Petrolatum groups in pain score on POD 14. ^*^p < 0.05 for RAO group vs. MEBO and Petrolatum groups in exudate score on POD 14.

### 3.2 Comparison of wound healing duration and wound healing rate

As shown in **Table 3**, the RAO group demonstrated a significantly shorter wound healing duration and a significantly higher wound healing rate compared with the MEBO and petrolatum groups. The differences among the groups were statistically significant (both p < 0.05).

**Table 3:** Comparison of wound healing duration and wound healing rate among the three groups.

Group	*n*	Wound healing duration (days)	Wound healing rate (%)		
			POD 14	POD 21	POD 28
RAO	30	27.60 ± 2.80^#^	32.46 ± 0.70	57.61 ± 0.78	83.53 ± 0.83
MEBO	30	29.83 ± 3.22^#^	30.29 ± 0.57	54.66 ± 1.13	79.37 ± 1.16
Petrolatum	30	32.10 ± 3.83^#^	27.94 ± 0.50^*^	44.18 ± 0.96^*^	70.16 ± 0.88^*^
F/X^2^		13.879	14.099	52.775	49.401
*P*		0.000	0.000	0.000	0.000

^#^ *p* < 0.05 for RAO group vs. MEBO and Petrolatum groups in wound healing duration; ^*^p<0.05 for RAO group vs. MEBO group and Petrolatum group in wound healing rate.

### 3.3 Comparison of caspase-1, IL-1β, and IL-18 levels among the three groups

**Figure 1:**
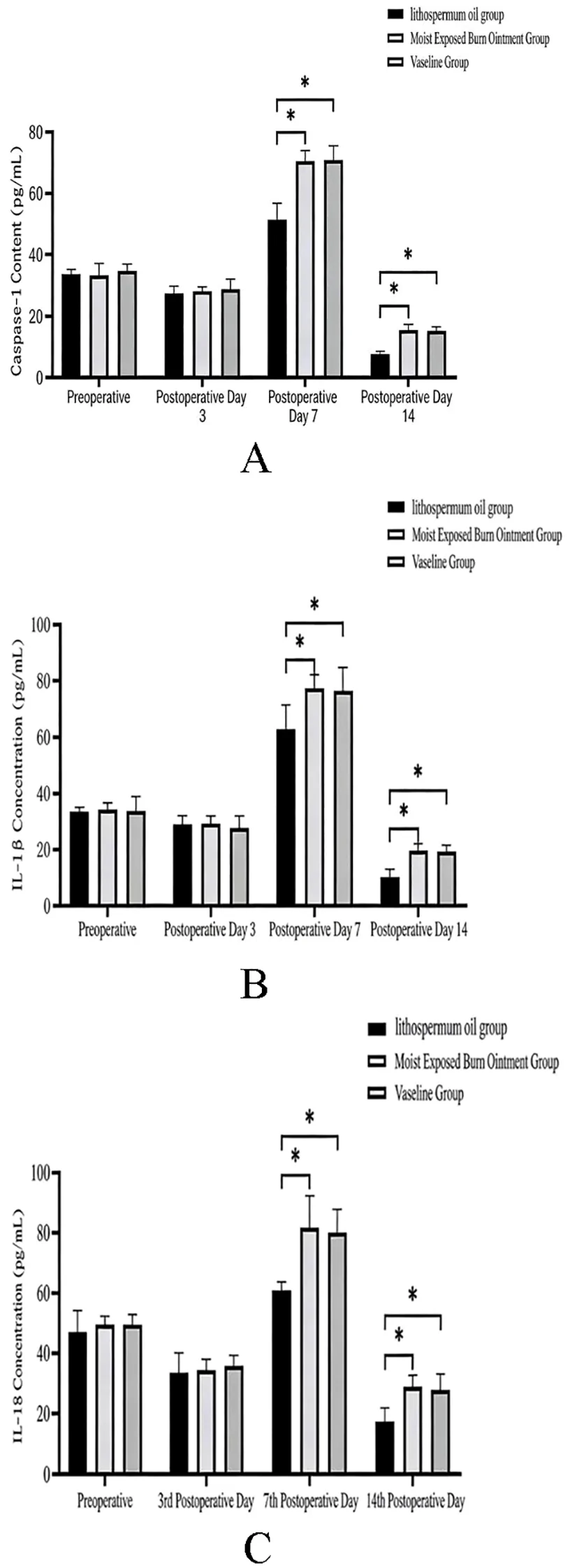
Comparison of caspase-1, IL-1β, and IL-18 protein levels in wound tissues among the three treatment groups. The * represents the comparison between the groups on the POD7 and POD14.

As shown in **Figure 1** and **Table 4**, elevated levels of the Caspase-1, IL-1β, and IL-18 were observed in wound tissue across all groups during the early postoperative period. The levels of these inflammatory mediators decreased on POD 3. As wound healing progressed, the expression levels in wound tissues increased and reached peak values on POD 7. Notably, the peak expression levels of these markers on POD 7 were lower in the RAO group than in the other groups, and the differences were statistically significant (all p < 0.05). By POD 14, the expression levels of these markers had decreased and stabilized at their lowest levels across all groups. The levels of Caspase-1, IL-1β, and IL-18 remained significantly lower in the RAO group than in the other two groups, with statistically significant differences observed (all p < 0.05).

**Table 4:** Comparison of Caspase-1, IL-1β, and IL-18 protein levels in wound tissues among the three groups (x̅ *± s*).

Group	Time point	Caspase-1	IL-1β	IL-18
RAO	Preoperative	33.54 ± 1.35	33.51 ± 1.45	47.15 ± 7.30
	POD 3	27.25 ± 2.35	28.83 ± 3.29	33.16 ± 6.75
	POD 7	51.25 ± 5.28	62.62 ± 8.60	61.41 ± 2.74
	POD 14	7.50 ± 0.78	10.21 ± 2.87	16.95 ± 4.47
MEBO	Preoperative	33.01 ± 3.96	34.01 ± 2.55	49.69 ± 2.74
	POD 3	28.10 ± 1.28	29.10 ± 2.89	34.08 ± 3.89
	POD 7	70.28 ± 3.45	77.09 ± 5.01	81.77 ± 10.67
	POD 14	15.35 ± 1.82	19.82 ± 2.22	28.19 ± 3.95
Petrolatum	Preoperative	34.73 ± 2.07	33.79 ± 5.15	49.42 ± 3.80
	POD 3	28.53 ± 3.36	27.63 ± 4.29	35.53 ± 3.56
	POD 7	70.68 ± 4.59	76.45 ± 8.34	80.26 ± 7.72
	POD 14	15.18 ± 1.22	19.45 ± 2.17	27.32 ± 5.26
F		55.858	24.862	9.260
*P*		0.000	0.000	0.004

Quantitatively, on POD 14, the mean levels of Caspase-1, IL-1β, and IL-18 in the RAO group were approximately 50.8%, 48.5%, and 39.9% lower, respectively, than those in the MEBO and petrolatum groups (calculated based on the data in **Table 4**). More importantly, these values in the RAO group had returned to near-preoperative baseline levels (Caspase-1: 7.50 ± 0.78 vs. 33.54 ± 1.35; IL-1β: 10.21 ± 2.87 vs. 33.51 ± 1.45; IL-18: 16.95±4.47 vs. 47.15±7.30), whereas the control groups remained at significantly elevated levels. This normalization of the local inflammatory milieu in the RAO group was temporally associated with the most significant improvements in pain, exudation, and healing rates observed on the same postoperative day, suggesting that the magnitude of these molecular reductions is directly linked to tangible clinical recovery.

### 3.4 IHC findings in wound tissues

**Figure 2:**
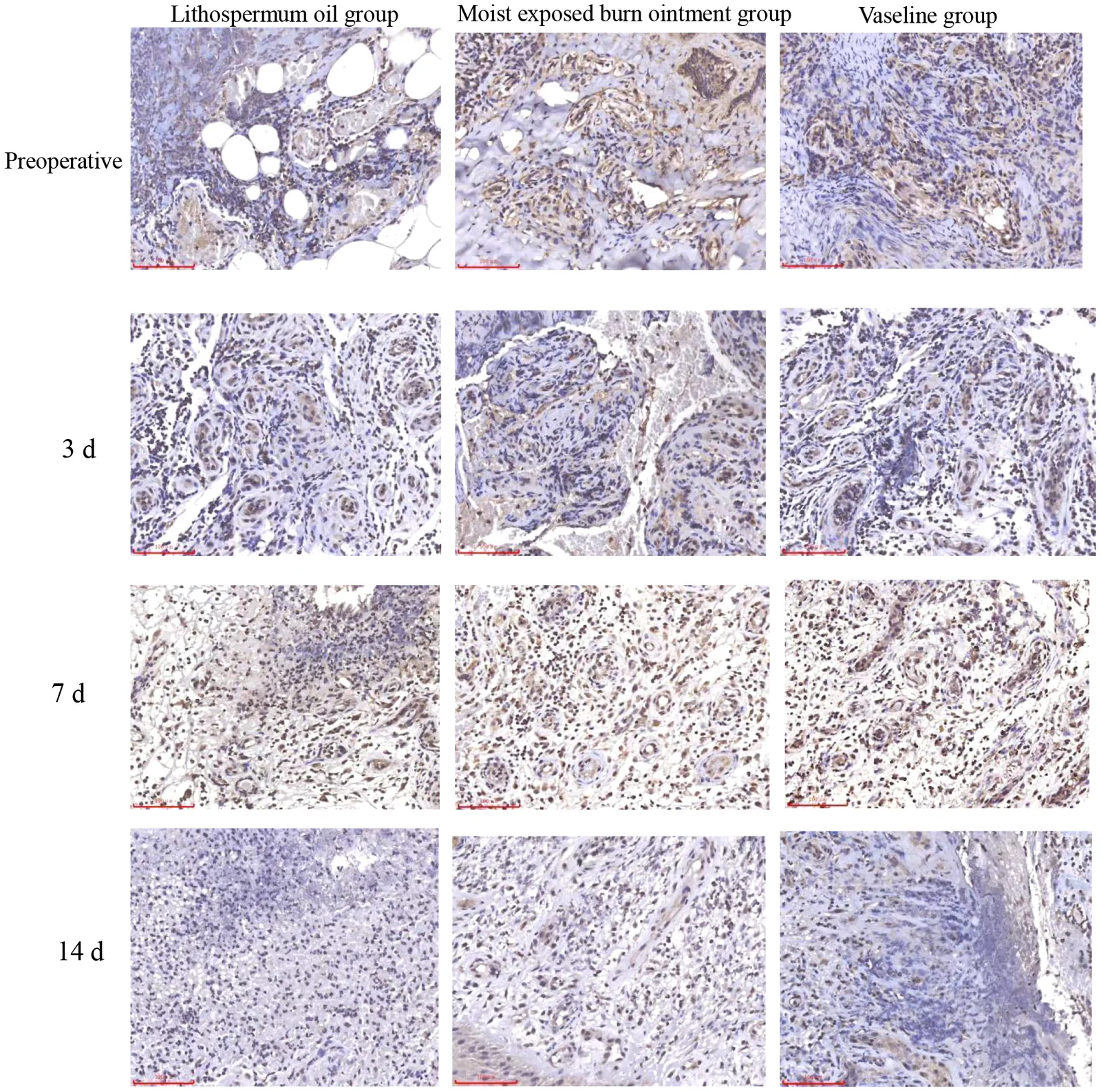
Immunohistochemical staining for caspase-1 in wound tissue from the three treatment groups (original magnification ×200).

**Figure 3:**
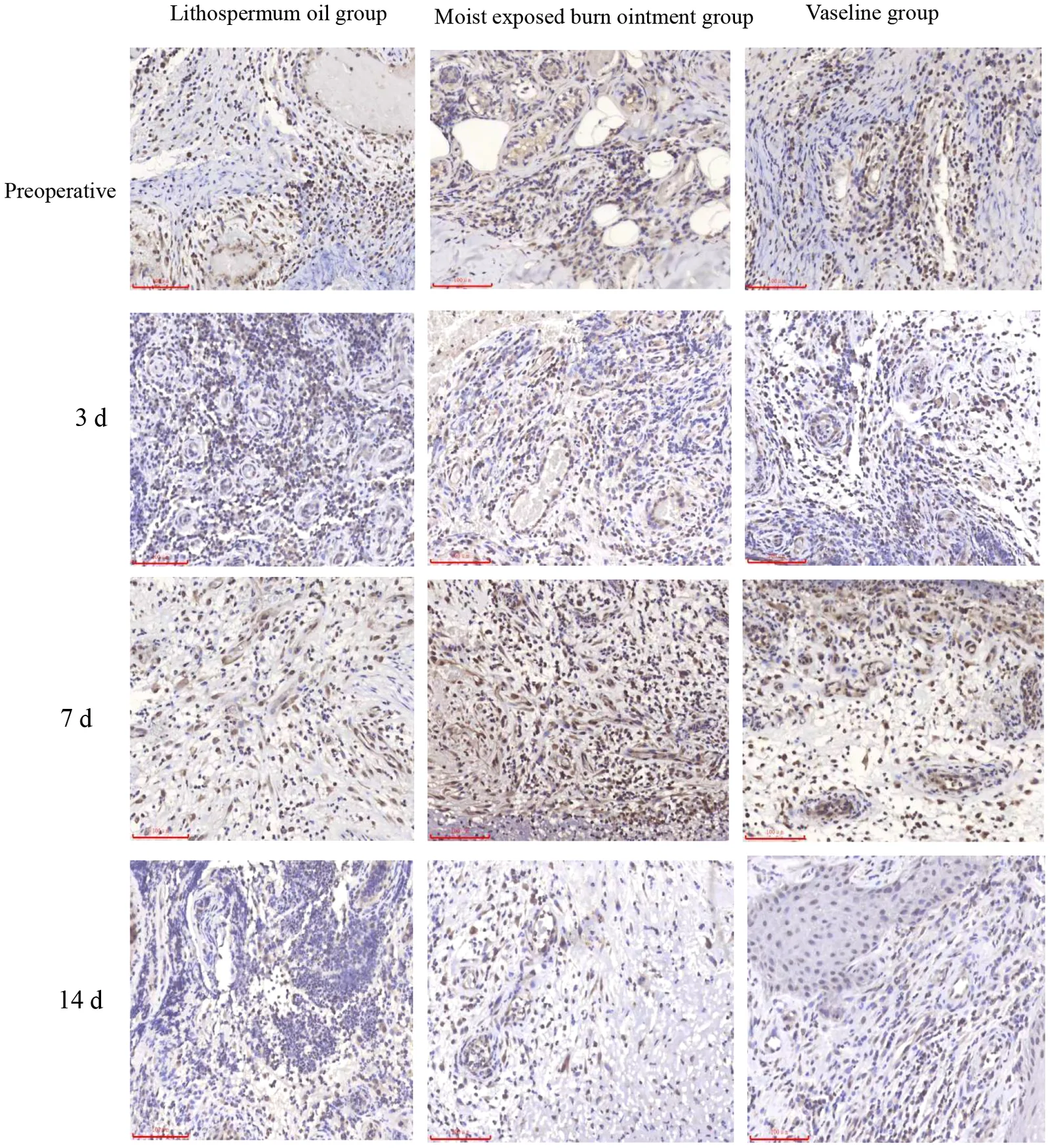
Immunohistochemical staining for IL-1β in wound tissue from the three treatment groups (original magnification ×200).

**Figure 4:**
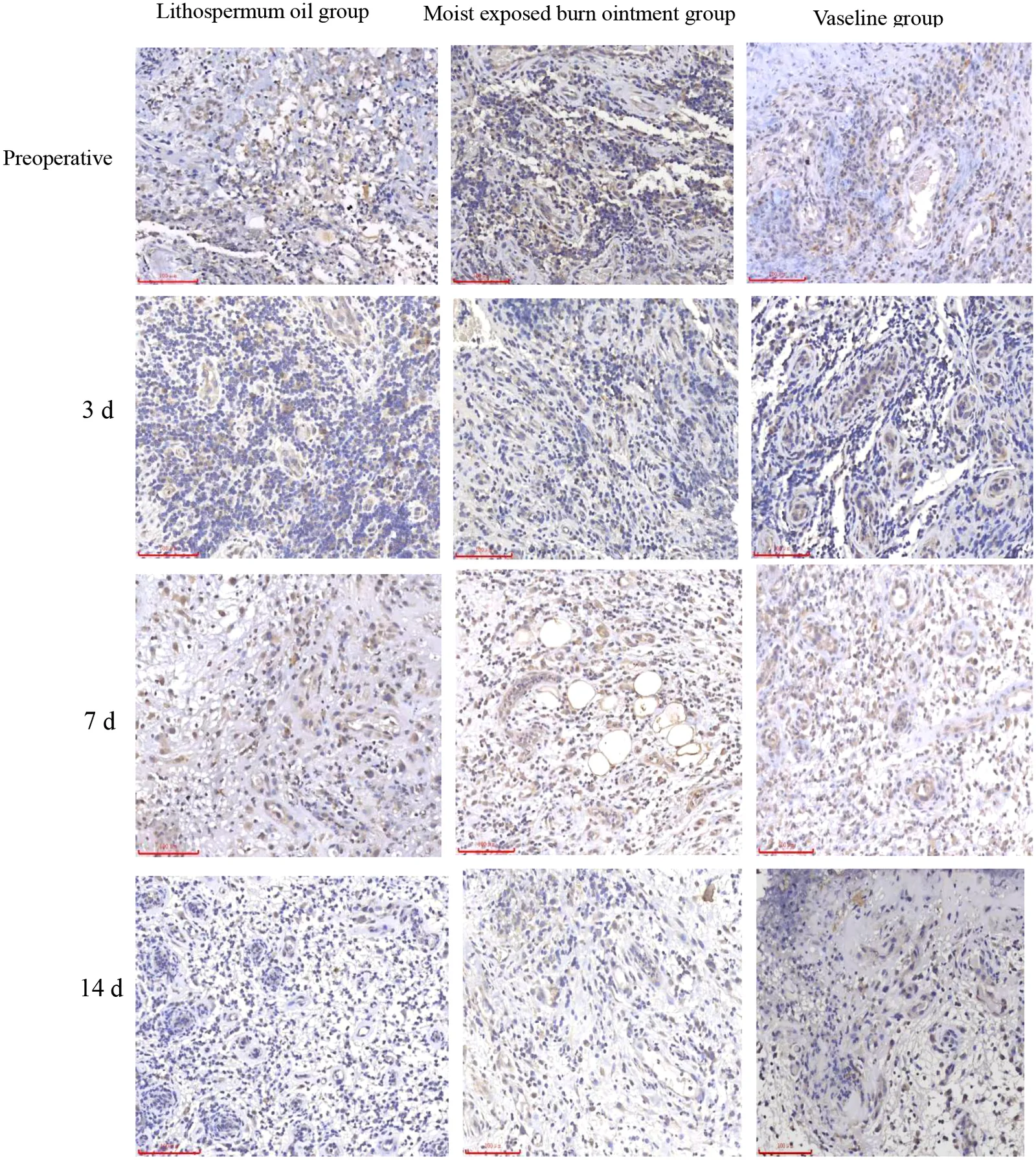
Immunohistochemical staining for IL-18 in wound tissue from the three treatment groups (original magnification ×200).

Representative IHC staining results are shown in **Figures 2**–**4**. Consistent with the ELISA findings, positive staining for Caspase-1, IL-1β, and IL-18 was observed in wound tissues from all groups prior during the postoperative healing period. Staining intensity decreased on POD 3 and increased to peak levels on POD 7. On POD 7, staining intensity for Caspase-1, IL-1β, and IL-18 was lower in the RAO group than in the MEBO and petrolatum groups. By POD 14, staining intensity had decreased further in all groups and was lowest in the RAO group. Quantitative analysis demonstrated significantly lower expression in the RAO group than in the other two groups, and the differences were statistically significant (all p < 0.05).

## 4 Discussion

According to TCM theory, although the primary lesion is removed following surgical treatment of anorectal abscess, residual heat-toxin may remain and contribute to the depletion of qi and blood, resulting in inadequate nourishment of the muscles and sinews and consequently delayed granulation tissue regeneration. External therapy is a distinctive treatment modality in TCM that involves the direct application of medicinal preparations to the body surface, meridians, collaterals, or affected areas for disease prevention and treatment (Shang et al., 2024). As stated in Yi Xue Ru Men (Introduction to Medicine) states, “If putrid tissue is not removed, new flesh will not grow,” and “If putrid tissue is not thoroughly eliminated, new tissue may grow either aberrantly or not at all.” Within TCM theory, the removal of putrid tissue is regarded as a prerequisite for tissue regeneration, and normal granulation tissue formation is considered to occur only after the wound surface has been completely cleared of putrid and turbid substances (Lu et al., 2021). Previous studies have proposed that Chinese herbal medicines can facilitate the removal of putrid and turbid substances from the wound surface, thereby contributing to tissue repair and regeneration. The underlying mechanism may be closely associated with the downregulation of inflammatory cytokines and the upregulation of factors related to wound healing (Zhu et al., 2016). Therefore, the TCM therapeutic principle of removing putrid tissue and promoting granulation has been widely applied in the management of chronic wounds.

RAO is a proprietary preparation routinely used at the study institution and is considered to possess the functions of clearing heat and draining dampness, relieving pain, arresting bleeding, resolving blood stasis, and promoting granulation. In this formulation, Radix Arnebiae serves as the sovereign herb and is used to clear heat, cool the blood, activate blood circulation, and detoxify. It is commonly applied in the treatment of carbuncles, ulcers, eczema, burns, and scalds. Rhizoma Coptidis and Radix et Rhizoma Rhei serve as minister herbs. Rhizoma Coptidis is traditionally used to clear heat, dry dampness, purge fire, and detoxify, whereas Radix et Rhizoma Rhei is used to clear heat, detoxify, activate blood circulation, and disperse blood stasis. As a minister drug, it not only reinforces the functions of clearing heat and draining dampness of the sovereign drug but also contributes to activating blood circulation, resolving stasis, and promoting tissue regeneration.

In combination, these herbs are considered to enhance the functions of clearing heat and draining dampness while facilitating blood circulation and reducing swelling. Gypsum Fibrosum is used to clear heat, purge fire, astringe sores, and promote granulation. Rhizoma Bletillae is used to astringe and arrest bleeding, reduce swelling, and promote granulation. Borneolum Syntheticum is used to clear heat, relieve pain, dissipate nodules, and reduce swelling. These three agents serve as assistant and courier herbs. Along with reinforcing the functions of clearing heat and draining fire of the sovereign herb, they act synergistically to reduce swelling and promote healing.

According to TCM theory, the pathological characteristics of the postoperative state following incision and drainage of perianal abscess are primarily attributed to exuberant heat-toxin, qi stagnation, and blood stasis. Consequently, RAO is considered to address these pathological processes through the principles of clearing heat and draining dampness, relieving pain, arresting bleeding, resolving blood stasis, and promoting granulation.

Pyroptosis is a form of programmed cell death characterized by inflammatory features and has been implicated in the pathogenesis of inflammatory and infectious diseases. It is characterized by cellular swelling, plasma membrane pore formation, membrane rupture, and the release of intracellular contents, resulting in amplification of inflammatory responses (Wei et al., 2022). Pyroptosis is referred to as inflammatory necrosis. It is typically initiated by inflammasomes and represents a form of cell death with inflammatory characteristics. Among the inflammasomes, the NLRP3 inflammasome is recognized as an important regulator of pyroptotic signaling pathways (Gaul et al., 2021; Wang et al., 2023).

Activation of inflammasomes promotes the activation of caspase-1 through interactions with adaptor proteins containing caspase recruitment domains. Activated caspase-1 cleaves the precursor forms of IL-1β and IL-18, generating mature cytokines that are released into the extracellular environment and contribute to inflammatory cell recruitment and propagation of inflammatory responses (Yu et al., 2021). IL-1β is involved in the regulation of neutrophil activation and recruitment of monocytes and macrophages and can further influence downstream cytokine signaling networks, including pathways involving IL-18 (Lv et al., 2023; Feng et al., 2025; Thangaveloo et al., 2025). Previous studies have demonstrated that caspase-1 also cleaves gasdermin D (GSDMD), generating the active N-terminal fragment (GSDMD-N)which forms pores in the plasma membrane and facilitates the release of IL-1β and IL-18, representing a key molecular event in pyroptotic cell death (Wu et al., 2018). Therefore, caspase-1 is considered a central mediator of pyroptosis-related signaling pathways.

We acknowledge several limitations in this study. First, although we observed that RAO downregulates pyroptosis-related mediators (caspase-1, IL-1β, and IL-18) during wound healing, we did not assess macrophage polarization markers (M1/M2), including iNOS, TNF-α, and CD86 for M1, or Arg-1, CD206, and IL-10 for M2; given that macrophages are central to the transition from inflammation to proliferation/repair, evaluation of these markers would have offered deeper insight into the local immune microenvironment. Second, the potential crosstalk between pyroptosis and macrophage polarization remains unexplored in our current dataset; future work will incorporate multiplex cytokine assays and immunohistochemical staining for M1/M2 surface markers to better clarify the integrated immunological pathways through which RAO facilitates wound repair. Third, while petrolatum serves as a clinically relevant and practical blank control in real-world wound care, its use as the control group does not enable us to dissociate the specific effects of RAO’s active ingredients from the protective influence of the vehicle itself; this limitation warrants future vehicle-controlled studies using a matched base preparation to isolate and quantify the active components’ contributions. Fourth, we did not include formal patient-reported outcome (PRO) measures—such as validated quality-of-life questionnaires (e.g., SF-36 or EQ-5D), satisfaction surveys, or objectively documented return-to-work time—as these were not pre-specified in our original protocol, which focused on objective healing parameters and molecular mechanisms; although retrospective collection would be methodologically unreliable due to recall bias, we recognize that PROs are an important dimension of clinical efficacy. Nonetheless, the significant improvements in observer-assessed pain scores, wound exudation, and healing duration—all of which directly affect patient comfort, daily activities, and functional recovery—provide strong surrogate evidence of clinically meaningful benefit, and future larger-scale studies should include validated PRO instruments to further substantiate the patient-centered value of RAO. Finally, a major mechanistic limitation deserves emphasis: the reduced levels of caspase-1, IL-1β, and IL-18 in the RAO group demonstrate a statistical association between RAO treatment and attenuated pyroptosis-related markers, but our clinical *in vivo* data cannot establish a direct causal relationship between specific RAO components and NLRP3/caspase-1 inhibition; RAO is a complex mixture of multiple herbal extracts, and we have not identified which individual compounds—or which synergistic combinations—mediate the observed effects; moreover, the biomarker changes could be secondary to other RAO-mediated actions (e.g., improved wound perfusion, reduced bacterial colonization, or enhanced epithelialization) rather than a direct pharmacological blockade of the pyroptosis cascade. Therefore, while our findings offer a strong clinical rationale and a testable hypothesis, definitive proof that RAO directly suppresses NLRP3/caspase-1/GSDMD-mediated pyroptosis will require further validation through *in vitro* cellular assays (e.g., using specific NLRP3 inhibitors or caspase-1 knockout models) and *in vivo* animal studies with pathway-specific interventions. We have explicitly acknowledged this limitation in the revised Discussion to appropriately contextualize the speculative nature of the TCM–pyroptosis connection.

In the present study, postoperative pain and wound exudation improved over time in all treatment groups. Importantly, treatment with RAO was associated with a shorter wound healing duration and a higher wound healing rate, alongside significantly lower protein levels of caspase-1, IL-1β, and IL-18 in wound tissues on POD 14. Based on these correlational findings, we speculate that the beneficial effects of RAO on wound healing might be closely associated with the modulation of inflammatory mediators involved in pyroptosis-related pathways. However, it is critical to acknowledge that, as an *in vivo* clinical study, our data reveal statistical associations rather than direct causal mechanisms. RAO contains a complex mixture of bioactive herbal compounds, and its effects on pyroptosis could be indirect, possibly mediated through upstream inflammatory signaling or microenvironmental changes. Therefore, while our findings provide a preliminary clinical clue, definitive evidence that RAO directly suppresses pyroptosis (e.g., via the NLRP3/caspase-1/GSDMD axis) requires further validation through *in vitro* cellular assays and animal models using specific pathway inhibitors or genetic knockouts.

## Data Availability

The original contributions presented in the study are included in the article/supplementary material. Further inquiries can be directed to the corresponding author.
